# Transcriptional abundance is not the single force driving the evolution of bacterial proteins

**DOI:** 10.1186/1471-2148-13-162

**Published:** 2013-08-02

**Authors:** Wen Wei, Tao Zhang, Dan Lin, Zu-Jun Yang, Feng-Biao Guo

**Affiliations:** 1 Center of Bioinformatics and Key Laboratory for NeuroInformation of the Ministry of Education, School of Life Science and Technology, University of Electronic Science and Technology of China, 610054 Chengdu, China

**Keywords:** Evolutionary rates, Bacteria, Multiple features, Transcriptional abundance

## Abstract

**Background:**

Despite rapid progress in understanding the mechanisms that shape the evolution of proteins, the relative importance of various factors remain to be elucidated. In this study, we have assessed the effects of 16 different biological features on the evolutionary rates (ERs) of protein-coding sequences in bacterial genomes.

**Results:**

Our analysis of 18 bacterial species revealed new correlations between ERs and constraining factors. Previous studies have suggested that transcriptional abundance overwhelmingly constrains the evolution of yeast protein sequences. This transcriptional abundance leads to selection against misfolding or misinteractions. In this study we found that there was no single factor in determining the evolution of bacterial proteins. Not only transcriptional abundance (codon adaptation index and expression level), but also protein-protein associations (PPAs), essentiality (ESS), subcellular localization of cytoplasmic membrane (SLM), transmembrane helices (TMH) and hydropathicity score (HS) independently and significantly affected the ERs of bacterial proteins. In some species, PPA and ESS demonstrate higher correlations with ER than transcriptional abundance.

**Conclusions:**

Different forces drive the evolution of protein sequences in yeast and bacteria. In bacteria, the constraints are involved in avoiding a build-up of toxic molecules caused by misfolding/misinteraction (transcriptional abundance), while retaining important functions (ESS, PPA) and maintaining the cell membrane (SLM, TMH and HS). Each of these independently contributes to the variation in protein evolution.

## Background

Amino acid substitution rates vary considerably among different proteins. Although rapid progress has been made in determining the most important factors that shape protein evolution, the challenge remains to assess the relative importance of various variables, such as gene expression level, essentiality (ESS) and protein interactions [1-10]. One early study [11] proposed a negative correlation between the severity of gene knockout effects and coding sequence evolution, which was dependent upon the notion that purifying selection should be more efficient for essential genes than those that are non-essential. A link has been discovered between protein expression levels and evolutionary rates (ERs) in both unicellular and multicellular organisms [7,12-19].

In general, genes that are highly expressed preferentially use optimal codons to improve translational efficiency. The codon adaptation index (CAI), a measure of synonymous codon usage bias, has been widely used as a proxy for gene expression levels [20]. When CAI values were used as a substitute for actual expression levels in yeast [2] and bacteria [12], only a small proportion of rate variation in protein evolution can be explained by ESS. After replacing CAI values with experimental data and controlling for gene expression levels, ESS still had significant effects on protein ERs, but did not appear to be a major determinant of protein evolution [21,22]. CAI, expression level, and protein abundance can account for most of the variation in yeast protein ERs [13]. Keeping proteins from misfolding or misinteraction result in the slow evolution of highly expressed genes, and impose a general constraint on coding sequence evolution [7,23]. However, by using noiseless variables, protein interactions have explained more ER variation than transcriptional abundance [24]. Results from another study suggest that the molecular evolution of protein-coding genes is affected by both the context of extrinsic translational expression rates and intrinsic structural-functional constraints [25].

Despite the large number of studies evaluating the effects of various mechanisms during protein evolution, the relative importance of these factors compared with transcriptional abundance remains to be elucidated. In this study, we investigated the effects of various biological features on protein-coding sequence ERs for 18 bacterial species. The following genomic variables: CAI; experiment-based expression level (EL); ESS; number of protein-protein associations (PPA); mRNA folding strength (MFS); hydropathicity score (HS); aromaticity score (AS); protein length (LEN); replication strand bias (RSB); number of transmembrane helices (TMH); and subcellular localization [cytoplasm (SLC), cytoplasmic membrane (SLM), periplasm (SLP), outer membrane (SLO), extracellular (SLE), and cell wall (SLW)] have been summarized (Table 1).

**Table 1:** Bacterial features examined in this study

**Feature**	**Abbreviation**	**Type**	**Source**
Essentiality	ESS	binary	DEG
Evolutionary rate (Ka)	ER	real	PAML
Codon adaption index	CAI	real	Python script
Expression level	EL	real	GEO
mRNA folding strength	MFS	real	ViennaRNA
Number of protein–protein associations	PPA	integer	STRING
Subcellular localization: cytoplasm	SLC	real	PSORTb
Subcellular localization: cytoplasmic membrane	SLM	real	
Subcellular localization: periplasm	SLP	real	
Subcellular localization: outer membrane	SLO	real	
Subcellular localization: extracellular	SLE	real	
Subcellular localization: cell wall	SLW	real	
Number of transmembrane helices	TMH	integer	TMHMM
Hydropathicity score	HS	real	CodonW
Aromaticity score	AS	real	
Length of protein in amino acids	LEN	integer	
Replication strand bias	RSB	binary	DoriC

## Results and discussion

### Genomic feature correlates of protein ER

In this study, potential correlation between ER and 16 features (Table 1) for 18 bacterial species (Table 2) was investigated. We observed strong correlations among CAI, EL, PPA, SLC, and ER; and less strong but significant correlations among ESS, SLM, TMH, AS and ER. However, weak relationships were found among MFS, SLP, SLO, SLE, SLW, HS, LEN, RSB and ER.

**Table 2:** Bacterial species investigated in this study

**Organism**	**Abbreviation**	**Codon usage separation (CUS)**	**GC content**	**Generation times **[29]
*Acinetobacter *ADP1	Abay	0.845	40.4	0.5
*Bacillus subtilis *168	Bsub	0.869	43.5	0.43
*Bacteroides thetaiotaomicron *VPI-5482	Bthe	0.291	42.8	1.47
*Caulobacter crescentus *NA1000	Ccre	0.754	67.2	1.5
*Escherichia coli *K-12	Ecol	0.879	50.8	0.35
*Francisella novicida *U112	Fnov	0.339	32.5	3
*Haemophilus influenzae *Rd KW20	Hinf	0.877	38.2	0.5
*Helicobacter pylori *26695	Hpyl	0.148	38.9	2.4
*Mycoplasma genitalium *G37	Mgen	0.529	31.7	12
*Mycoplasma pulmoni*s UAB CTIP	Mpul	0.032	26.6	1.5
*Mycobacterium tuberculosis *H37Rv	Mtub	0.246	65.6	19
*Porphyromonas gingivalis *ATCC 33277	Pgin	0.800	48.4	2.7
*Pseudomonas aeruginosa *UCBPP-PA14	Paer	0.915	66.3	0.5
*Staphylococcus aureus *NCTC 8325	Saur	0.807	32.8	0.4
*Streptococcus pneumoniae *TIGR4	Spne	0.893	39.7	0.5
*Streptococcus sanguinis *SK36	Ssan	0.962	43.4	-
*Salmonella typhimurium *LT2	Styp	0.915	52.2	0.4
*Vibrio cholera *O1 biovar El Tor N16961	Vcho	0.897	47.4	0.2

#### ***Transcriptional abundance***

A single variable linked to transcriptional abundance (CAI, EL and protein abundance) was found to explain the dominance of observed variation in yeast ERs [13]. CAI and EL are related to transcriptional abundance, while protein abundance is a result of the combined consequences of transcription and translation. Recent studies observed that MFS was strong for more abundant proteins, resulting in stronger evolutionary constraints of more highly expressed proteins [26,27]. We used three variables (CAI, EL and MFS) to highlight the impact of transcriptional abundance on ER.

**Figure 1:**
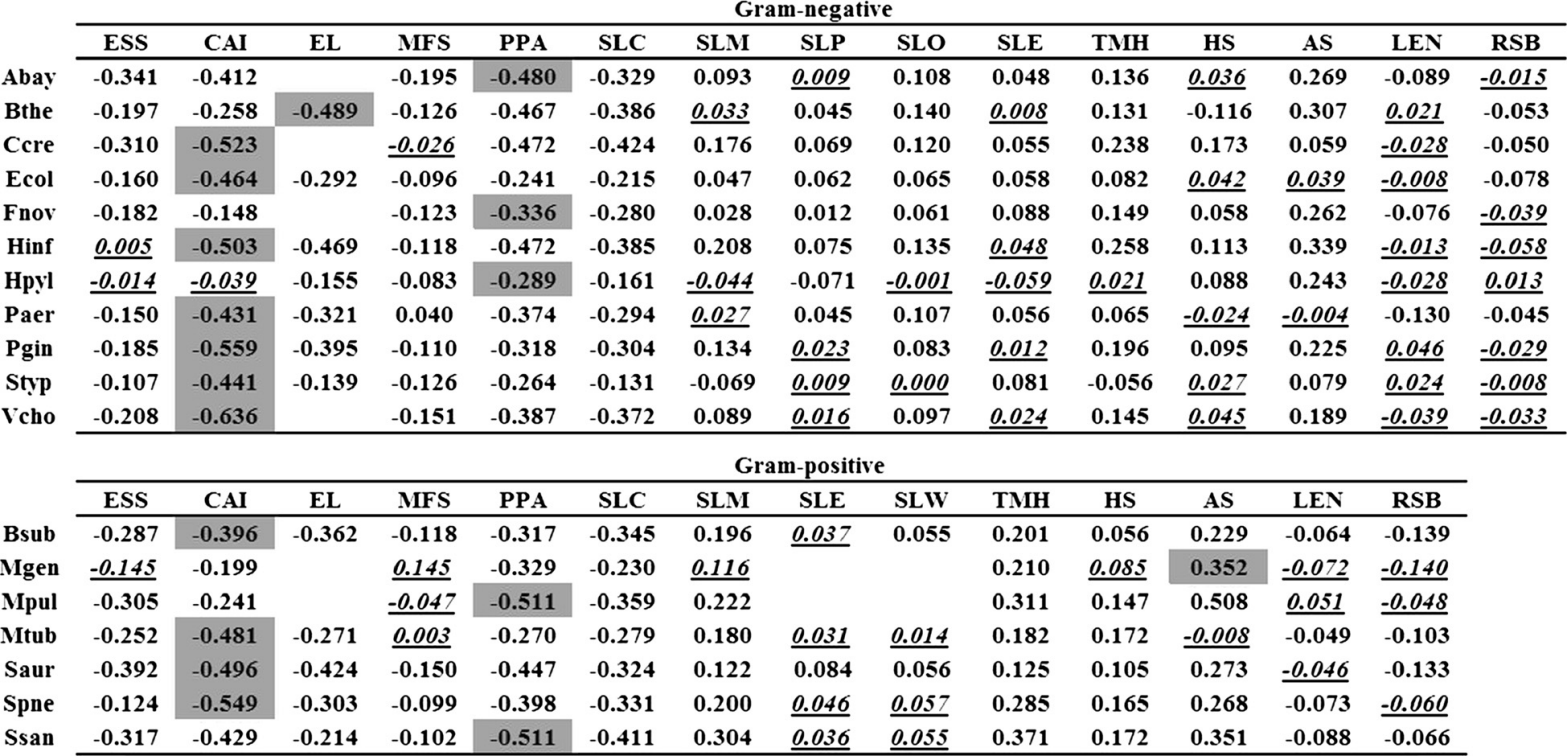
**Rank correlation coefficient of variables and expression rate (ER).** Gray cells indicate the highest absolute coefficients obtained for each bacterium. Blanks indicate that variables are non-existent or defective. Italicized and underlined values are non-significant at *p* > 0.05.

We used RNAfold (http://www.tbi.univie.ac.at/~ronny/RNA/) to predict the secondary structure of RNA and to compute the strength of mRNA folding. A recent study reported that RNAfold predicted MFS is moderately correlated with experimentally determined MFS [27]. Furthermore, it was also shown that the correlation of the computationally predicted MFS and ER was much weaker than that of the experimentally determined MFS and ER. Similarly, we also observed a low correlation between MFS predicted by RNAfold and ER in bacteria. However, in our study we found that CAI and EL were significantly linked to ERs for most bacteria, depending on rank correlation coefficients (Figure 1). The top absolute coefficients were dominated (12/18 bacterial species) by CAI-ER or EL-ER correlations. The CAI-ER coefficient in *Escherichia coli* was -0.464 (*p* = 5.45 × 10^-107^), which is greater than other coefficients. Of these bacteria, *Helicobacter pylori* showed no CAI-ER correlation (rho = -0.039, *p* > 0.05); this species was not subject to periods of competitive exponential growth [28]. As a result, there was a lack of translational selection related to codon usage. In an early study, codon selection for translation was observed to strongly correlate with growth rates [29]. We investigated the effects of growth on CAI-ER correlations, and found that weak correlations could be partially attributed to long-term bacterial generation times (Table 2).

**Figure 2:**
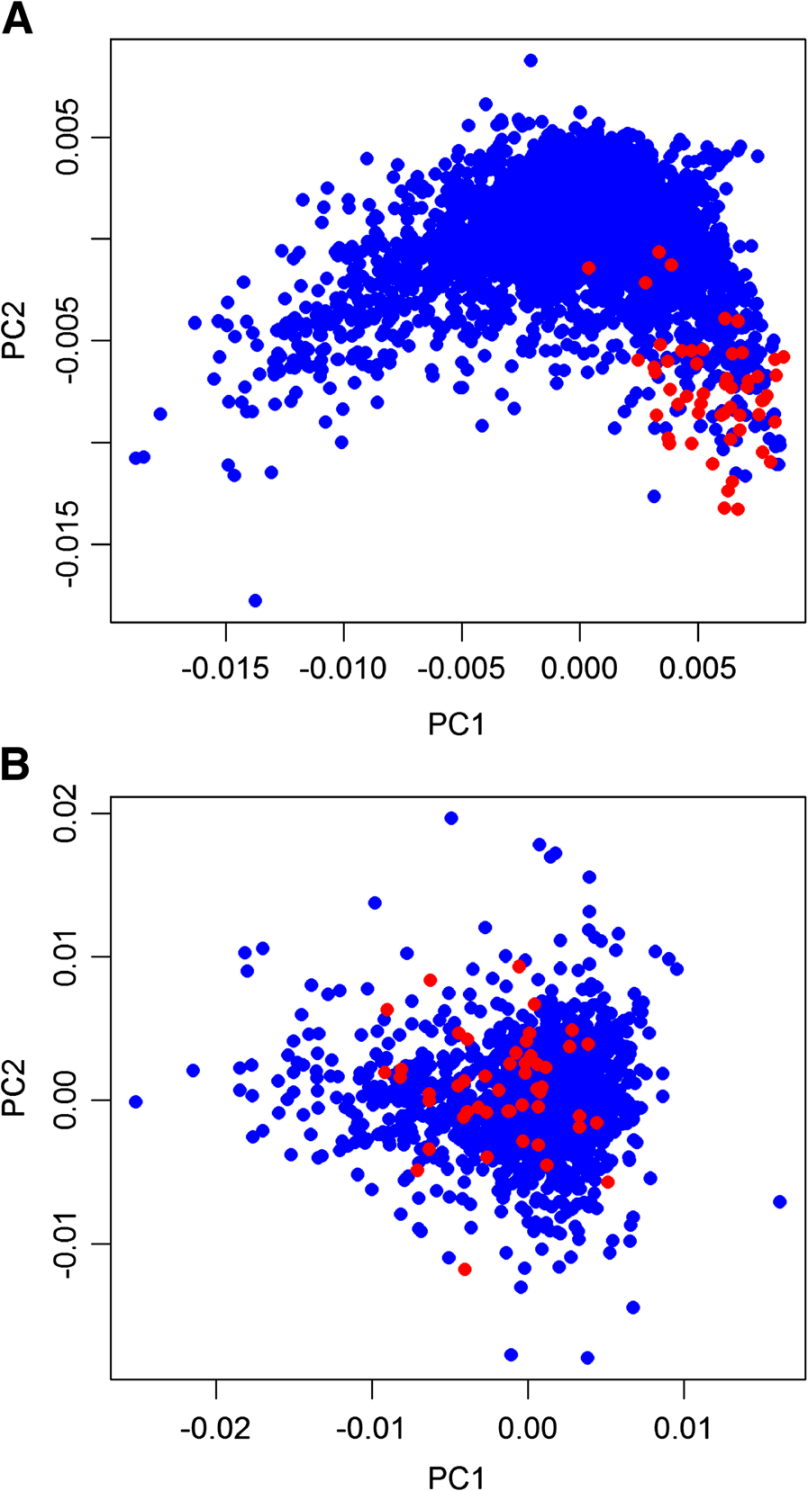
**Plots of correspondence analysis of relative synonymous codon usage (RSCU-CA).** PC1 is the first principal component and PC2 is the second one. Ribosomal and non-ribosomal genes are represented by red and blue dots, respectively. Significantly different codon usages were observed between ribosomal proteins and other proteins in the genome of **(A)***E. coli*, but were not observed in the genome of **(B)***H. pylori*.

To further investigate the impact of translational selection on CAI-ER correlation, codon usage separation (CUS) was used to measure the strength of codon bias. A greater CUS value indicated a stronger codon bias mediated by translational selection. We confirmed a correlation between CUS and the CAI-ER coefficient (Pearson’s r = -0.730, *p* = 5.83 × 10^-4^). Significantly different codon usage (CUS = 0.879) was found between ribosomal proteins and other proteins in the *E. coli* genome (Figure 2A); however, this was not observed in the *H. pylori* genome (CUS = 0.148; Figure 2B). The degree to which transcriptional abundance influences ERs correlated with the strength of translational selection.

It has been previously demonstrated that translational selection across species is also strongly affected by genomic GC content [30]. We found that CAI-ER coefficients significantly correlated with GC content (Pearson’s r = -0.473; *p* = 0.045). CAI-ER coefficients of GC-rich bacteria are significantly greater than those for AT-rich bacteria, as translational selection is often absent in AT-rich organisms [31]. It is also known that mRNAs have a stronger secondary structure if there are more GC-rich codons [32,33]. Moreover, there is stronger selection to improve translation efficiency for weak folding at translation-initiation sites of a gene in GC-rich hosts [34]. These GC-rich organisms preferentially use GC-rich optimal codons [35]. GC-rich genomes therefore show stronger translational selection compared with AT-rich genomes. Accordingly, we found that transcriptional abundance does not always influence ERs as GC content varies across species.

A recent study found that CAI, microarray-based EL or sequencing-based EL approaches for measuring transcriptional abundance affected the assessment of the importance of transcriptional abundance to ER [9]. We found that bacteria, whose EL-ER correlation was weaker than the CAI-ER correlation, demonstrated greater CUS (0.816 *vs.* 0.220). For those species strongly mediated by translational selection, CAI as opposed to EL likely better explains the variation of ER. Although RNA sequencing (RNA-seq) data could be more accurate than microarray data, there is currently little RNA-seq data available for most bacterial species. In this study, we derived EL from RNA-seq data for *E. coli*, and used microarray data for other bacterial species. Although the sequencing-based EL-ER correlation is weaker than the CAI-ER correlation in *E. coli*, it is stronger than other correlations. With the development of RNA-seq experiments, we believe that the assessment of EL-ER correlations could be more accurate, and the impact of EL on ER could be stronger in certain bacterial species. To compensate for the inadequacy of each single variable to represent expression levels, we used CAI, EL and MFS to describe the impact of transcriptional abundance on ERs.

#### ***Functional importance***

In an earlier study, it was proposed that ESS and protein interactions were negatively correlated with coding sequence ERs because of the constraints of important physical functions [3,6,11]. We used many types of protein associations (PPA), not only physical protein interactions (PPI), which were directly extracted from the STRING database. As expected, significant correlations between PPA/ESS and ERs were found for almost all the bacteria we investigated in our study. The strength of PPA-ER correlations was even greater than that of CAI-ER/EL-ER correlations in six organisms: *Acinetobacter* ADP1; *Francisella novicida*; *H. pylori*; *Mycoplasma genitalium*; *Mycoplasma pulmonis*; and *Streptococcus sanguinis*. In *F. novicida*, the ESS-ER correlation was also larger than that for CAI/EL-ER. The function of a gene is indeed an important driving force in bacterial protein evolution.

#### ***Variation in subcellular localization***

Most cellular activities, including many metabolic pathways and processes, occur within the SLC. In this study, we observed significant negative correlations between SLC and ER. For example, the correlation coefficient for *Caulobacter crescentus* was -0.424 (*p* = 5.18 × 10^-118^). The SLM surrounds the cytoplasm of living cells, and positive correlations between SLM and ER were also observed in our study. The cell membrane functions as a selective filter, allowing molecules either to be pumped across the membrane by transmembrane transporters, or to be diffused through protein channels. These transmembrane proteins are usually specific; as a consequence, SLM proteins are fast-evolving and well adapted. We also found, as expected, that TMH positively correlated with bacterial protein ERs. The positive correlations are relatively weak between other subcellular localizations (SLP, SLO, SLE, and SLW) and the ERs of proteins. Secreted proteins located in SLO/SLE for Proteobacteria and SLW/SLE for Firmicutes were found to rapidly evolve [10]. This could be a potential explanation of why SLW, SLO and SLE rapidly evolve.

#### ***Limitations of aromatic amino acids***

To manufacture proteins, microorganisms must synthesize their aromatic amino acids *via* the shikimate pathway. These amino acids have a limited source that impacts upon the rate at which translation errors can be corrected, and the maintenance of translation efficiency and accuracy. Therefore, the adoption of aromatic amino acids in functional or abundant proteins is not encouraged. In this study, we found that slowly evolving proteins tend to avoid adopting aromatic amino acids. In most of the investigated bacteria, AS positively and significantly correlated with ER (Figure 1).

#### ***Head-on conflict***

In many bacteria, genes tend to be encoded on the leading strand. The likelihood of a gene being found on the leading strand was weakly, but significantly, associated with ER in most of the studied bacteria. As an example, RSB of *Bacillus subtilis*, whose genome contains over 70% leading proteins, was significantly and positively correlated with ER (Pearson’s r = -0.139; *p* = 7.55 × 10^-11^). Transcription and replication occur simultaneously in bacterial cells [36-38]. Replication progresses much faster than transcription, and inevitable conflicts occur between DNA and RNA polymerases when they bind to the same template. Co-directional collisions occur when the leading strand is the template for transcription, resulting in head-on collisions taking place when the lagging strand is the template. Head-on collisions have particularly deleterious effects, as replication forks may be arrested and transcription slowed. Over the course of evolution, transcripts are more likely to be retained if they are on the leading strand, which explains why bacterial genes on the leading strand evolve more slowly than those on the lagging strand.

### Multiple factors cooperatively dominate ER

**Figure 3:**
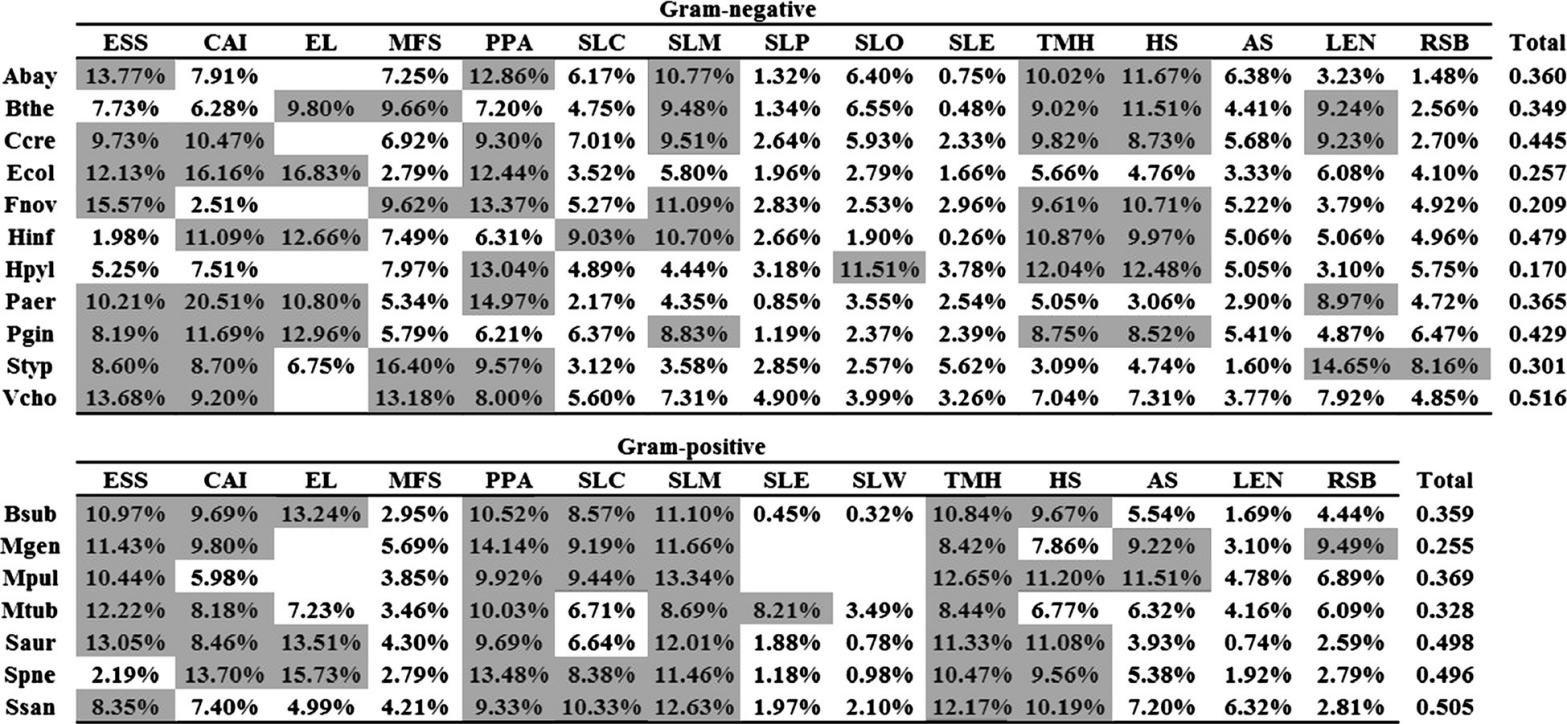
**Proportion of each independent contribution (R**^**2**^**) to total contributions.** Cells highlighted in gray indicate variables that contributed at least 8% of the total contributions to the indicated species. Blanks indicate that variables are non-existent or defective.

In both *E. coli* and *B. subtilis*, CAI has been identified as the most important driving force constraining ERs, through the use of partial correlation and multivariate regression analyses [12]. Drummond et al. first used principal component regression (PCR) analysis, and explained the dominant proportion of variation in yeast protein ERs by transcriptional abundance [13]. This analysis circumvents the problems of partial correlation and multivariate regression, as all principal components are orthogonal and independent. Therefore, it was useful to determine the independent contributions (R^2^) of biological features to yeast ERs [13]. In contrast to the situation reported for yeast, *E. coli* and *B. subtilis*[12,13], our PCR results suggest that the contributions of multiple factors are comparable for the determination of bacterial protein evolution (Figure 3). We found that *Staphylococcus aureus* was strongly influenced by codon bias (CUS = 0.915), with ESS, CAI, EL, PPA, SLM, TMH and HS representing 13.1, 8.5, 13.5, 9.7, 12.0, 11.3 and 11.1% of the total rate variation, respectively. These variables are comparable and account for 79.13% of the total variation. In other words, CAI and EL lose their dominance in explaining bacterial protein evolution, even in bacterial genomes with strong codon bias. Of these investigated factors, ESS, CAI, EL, PPA, SLM, THM and HS each represent over 8% of the total variation in 78 (14/18), 67 (12/18), 67 (7/12), 83 (15/18), 72 (13/18), 78 (14/18) and 67 (12/18), respectively, of bacterial genomes.

**Figure 4:**
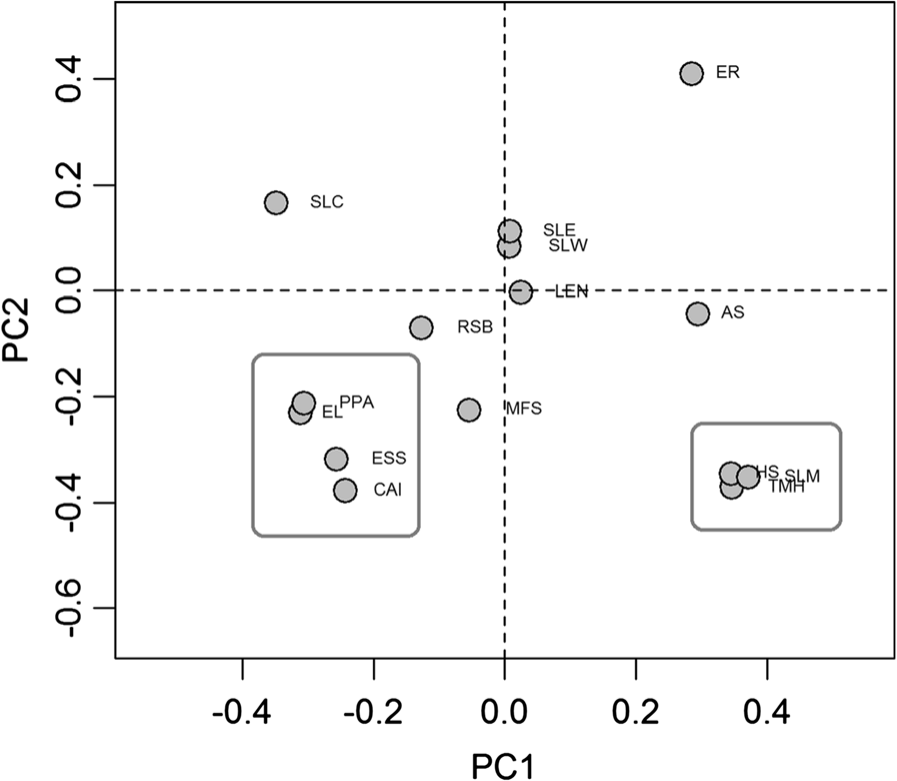
**Principal component plot of *S. aureus.*** Functional factors (ESS and PPA) and transcriptional abundance (CAI and EL) grouped together and strongly contributed to the PC2 that is opposite to that of the ER. Similarity, SLM, TMH and HS cooperatively and negatively affected the ER at the PC2.

Based on Correspondence Analysis (CA) results, we observed the universal rule that functional factors (ESS and PPA) and transcriptional abundance (CAI and EL) were roughly grouped together, opposing the ERs in the second principal component (PC2, see Methods) (Additional file 1: Figure S1, Figure 4). Evolutionary constraints on highly transcribed proteins might prevent misfolding [7] or misinteraction [23]. This can hamper functionality and even potentially produce a large quantity of toxic proteins. In contrast, constraints on essential or high connectivity genes possibly operate to avoid the abrogation of important physiological functions. The need for translational accuracy and robustness can help explain the selection exerted on ESS, PPA, CAI, and EL. In a principal component plot obtained for yeast, ESS and PPI were distant from CAI and EL [39]. This suggests a close link between functional factors and transcriptional abundance in some bacteria that is probably dependent on ER in some way.

In all the bacterial species we investigated, SLM, TMH and HS were found to cooperatively affect ER (Additional file 1: Figure S1). These three factors have been grouped in principal component plots (Figure 4). Membrane protein transport takes place *via* helix-dependent protein channels embedded in cell membranes, because of their hydrophobic structure. The need to maintain transmembrane protein function may help explain the relationship among SLM, TMH, and HS.

### Different forces drive ER in different species

According to PCR analysis, factors associated with transcriptional abundance (CAI, EL), important functionality (ESS, PPA) and transmembrane protein function (SLM, TMH, and HS) were the main contributors (8%) to protein ER variation in over 50% of bacterial species we studied. Transcriptional abundance is the most dominant factor in yeast [13], but not in mice [40] or bacteria (this study). The extent to which transcriptional abundance affects ERs correlates with the strength of codon bias. Our PCR analysis indicated multiple factors contribute to the rate of protein evolution in bacteria. We also found that PPA was a common important contributor to bacterial evolution, with greater effects than CAI/EL. Our results were basically identical to those presented by Plotkin and Fraser [24]-PPI appears to be responsible for most of the ER variation in yeast. The deleterious effects of protein misinteractions can affect the optimal protein concentrations and shape functional interaction networks [41]. Therefore there is a need to maintain proper interactions among high connectivity proteins as it constrains their evolution. Although ESS does not contribute strongly to yeast ERs, it is still an important factor in determining bacterial protein evolution. Our findings suggest that various forces drive protein sequence evolution in different species.

## Conclusions

We have uncovered new relationships among ERs in bacterial genomes related to protein subcellular localization, transmembrane helices, hydropathicity, aromaticity, and replication strand localization. ER had a significant negative correlation with SLC, but a significant positive correlation with SLM. Because of the effects of TMH and HS on SLM, these two variables were also found to positively, although relatively weakly, correlate with bacterial protein ERs. The impact of bacterial SLM/TMH/HS and SLC on ER is independent of functional importance and transcriptional abundance. This is consistent with results from a recent study in mammalian proteins [8]. We also found that proteins that evolved slowly in bacterial genomes tended to avoid adopting aromatic amino acids. Additionally, bacterial genes on the leading strand evolved more slowly than those with genes on the lagging strand. We investigated the independent contributions of biological features to ER, and found that the dominant effect of transcriptional abundance on ER is absent in bacteria. Factors that retain important functionality (ESS, PPA), maintain cell membrane function (SLM, TMH, and HS) and avoid a build-up of toxic molecules caused by misfolding or misinteraction (CAI, EL) influence the ERs of bacterial proteins. If more RNA-Seq data are available in the future, the correlation of EL-ER could be found to be stronger in certain bacterial species than reported here. However, the influences of PPA, ESS, SLM, TMH, and HS on ER are comparable with the impact of transcriptional abundance on ER in most bacteria.

## Methods

### Genomic features

#### ***Essentiality***

We investigated 18 bacterial species (Table 2) in the current version (7.0) of the Database of Essential Genes (DEG; http://tubic.tju.edu.cn/deg/), which hosts records of available essential genes identified by well-known genome-wide experimental techniques from a range of organisms [42]. In each of these experiments, almost all genes were investigated for their ESS scores; therefore datasets were not biased or partial. Complete coding sequences of these bacteria and their gene ESS annotations were obtained from GenBank and DEG databases, respectively.

#### ***Evolutionary rates***

Orthologous gene pairs between each genome pair were identified based on reciprocal best hits using the Blastp program with criteria of *E* <10^-5^, 80% minimum residues that could be aligned, and 30% identity. Protein sequences encoded by identified orthologous gene pairs were aligned with ClustalW [43], and then back-translated into nucleotide sequences based on their original sequences. Numbers of substitutions per non-synonymous site (*K*_*a*_) were calculated following Yang’s definition using the PAML package with default parameters [44]. We retained all ortholog assignments coding for more than 30 amino acids, which were not acquired by horizontal transfer, as determined by the Horizontal Gene Transfer [45] (HGT-DB; http://genomes.urv.cat/HGT-DB/) and DarkHorse [46] (http://darkhorse.ucsd.edu/) databases. Values for ERs were log-transformed after addition of a small constant (0.001).

#### ***CAI, expression level and mRNA folding strength***

Transcriptional abundance was predicted from CAI, expression levels and mRNA folding strength. CAI is a species-dependent codon bias measurement that has been widely used as an empirical approach for gene expressivity, especially in microbial genomes [20]. With this methodology, dozens of ribosomal protein genes were chosen as a reference set of highly expressed genes for each genome. Our mRNA levels, derived from RNA-seq data for *E. coli* and microarray data for other species, under favorable environmental conditions were extracted from the Gene Expression Omnibus [47] (GEO; http://www.ncbi.nlm.nih.gov/geo/) database. Data were obtained for the following bacteria: *B. subtilis* (GEO Sample Accession Numbers GSM177105–GSM177118); *Bacteroides thetaiotaomicron* (GSM40897–GSM40906); *E. coli* (GSM99211–GSM99216); *Haemophilus influenzae* (GSM114031–GSM114033); *H. pylori* (GSM623401–GSM623404); *Mycobacterium tuberculosis* (GSM71958, GSM71988–GSM71990); *Porphyromonas gingivalis* (GSM590017); *Pseudomonas aeruginosa* (GSM462061–GSM462064, GSM462352–GSM462355); *S. aureus* (GSM724739–GSM724741), *Streptococcus pneumonia* (GSM673840); *Streptococcus sanguinis* (GSM908371–GSM908373); and *Salmonella typhimurium* (GSM874413–GSM874415). Expression level values were scaled using a logarithmic function.

The secondary structures of mRNAs, for a folding temperature under 30°C, were predicted by RNAfold within the ViennaRNA package [48]. Windows comprising 150 nucleotides were slid in 10 nucleotide steps during analysis [26]. At each nucleotide, the probability that it paired was estimated by the number of sliding windows with which it paired, divided by the number of sliding windows that include the nucleotide. We then used the average pairing possibility for an mRNA to estimate its folding strength.

#### ***Number of protein–protein associations***

Protein-protein association data were obtained from the STRING database [49] (http://string-db.org/). These association data included physical PPIs and other links such as co-expression data. From the original data, we computed the number of associations for each gene using a default confidence score cutoff of 0.4.

#### ***Subcellular localization and number of transmembrane helices***

We used PSORTb v3.0 [50] (http://www.psort.org/psortb/) to predict subcellular localization of proteins. Four subcellular localization types can be predicted for Gram-positive bacteria and five types can be predicted for Gram-negative bacteria. For a certain localization type, genes were assigned PSORTb prediction scores if they belong to this type, and 0 if they did not. The number of transmembrane helices was predicted from bacterial proteomes using the TMHMM Server v2.0 (http://www.cbs.dtu.dk/services/TMHMM/).

#### ***Protein hydropathicity, aromaticity, and length***

We used CodonW (http://codonw.sourceforge.net/) to determine hydropathicity, aromaticity and protein length. The general average hydropathicity score for each gene product was obtained by calculating the arithmetic mean of the sum of the hydropathic indices for each amino acid. Aromaticity scores are indices for indicating frequency of aromatic amino acids.

#### ***Replication strand bias***

Replication origin and terminus positions for each bacterial species were annotated using the DoriC database [51] (http://tubic.tju.edu.cn/doric/index.html). Genes were assigned a value of 1 if these positions were located on the leading strand, and 0 if otherwise.

### Statistical analysis

#### ***Spearman rank correlation and PCR***

Spearman’s rank correlation test was used to investigate expected direct correlations between each variable and ER. To further determine the independent contribution (R^2^) of each biological feature to ER, we used PCR.

#### ***CUS***

To assess the impact of translation selection on codon usage, we investigated differences in relative synonymous codon usage (RSCU) between ribosomal proteins and non-ribosomal proteins using correspondence analysis (RSCU-CA). Correspondence analysis is a classical technique to reduce the dimensionality of a dataset by transforming it into its principal components. The first principal component (PC1) maximizes the standard deviation of the derived variable, while the second principal component (PC2) maximizes the standard deviation among axes uncorrelated with the first. The PC1 and PC2 could effectively explain the original 64-D codon datasets. We observed from the PC1-PC2 plot the differential codon usage pattern between ribosomal proteins and non-ribosomal proteins. Then we defined the CUS of ribosomal proteins as the percentage of ribosomal proteins falling outside the non-ribosomal protein cluster on the PC1-PC2 plot. The reference range (90%) of non-ribosomal proteins on the plot was defined as:

(1) $$\begin{array}{ll} \bar{X}‒1.64S & <X<\bar{X}+1.64S;\;\bar{Y}‒1.64S<Y\\ & <\;\bar{Y}+1.64S \end{array}$$

where $\bar{X}$, $\bar{Y}$, and S denote the average PC1 and PC2 values of non-ribosomal proteins, and the standard deviation of the principal component value, respectively. A greater CUS indicates a greater difference in codon usage between ribosomal proteins and non-ribosomal proteins. All statistical analyses were conducted and plots generated using the R package (http://www.r-project.org/).

## Abbreviations

PCR: Principal component regression; RSCU: Relative synonymous codon usage; CA: Correspondence analysis; PC: Principal component; CUS: Codon usage separation; GEO: Gene expression omnibus; DEG: Database of essential genes.

## Competing interests

The authors declare that they have no competing interests.

## Authors’ contributions

WW participated in the design of the study, compilation of the python script, collection and preparation of data, performed the analyses, and drafted the majority of the manuscript. TZ assisted in writing the python code to implement Blast and PAML. DL double-checked the results. ZJY took part in drafting and editing the manuscript. FBG conceived and guided the study, and also assisted in writing the manuscript. All authors read and approved the final version of the manuscript.

## Supplementary Material

Additional file 1: Figure S1. Principal component plots of other 17 bacteria. Click here for file
