# Supplementary material for: Transcriptional abundance is not the single force driving the evolution of bacterial proteins
Source: BMC Evol Biol. 2013 Aug 2;13:162. doi: 10.1186/1471-2148-13-162 (PMC3734234; doi:10.1186/1471-2148-13-162)
Supplement: Additional file 1: Figure S1. — Principal component plots of other 17 bacteria. [file 1471-2148-13-162-S1.doc]

Supplementary Figure 1

| Gram-negative organism | | |
| --- | --- | --- |
| Abay  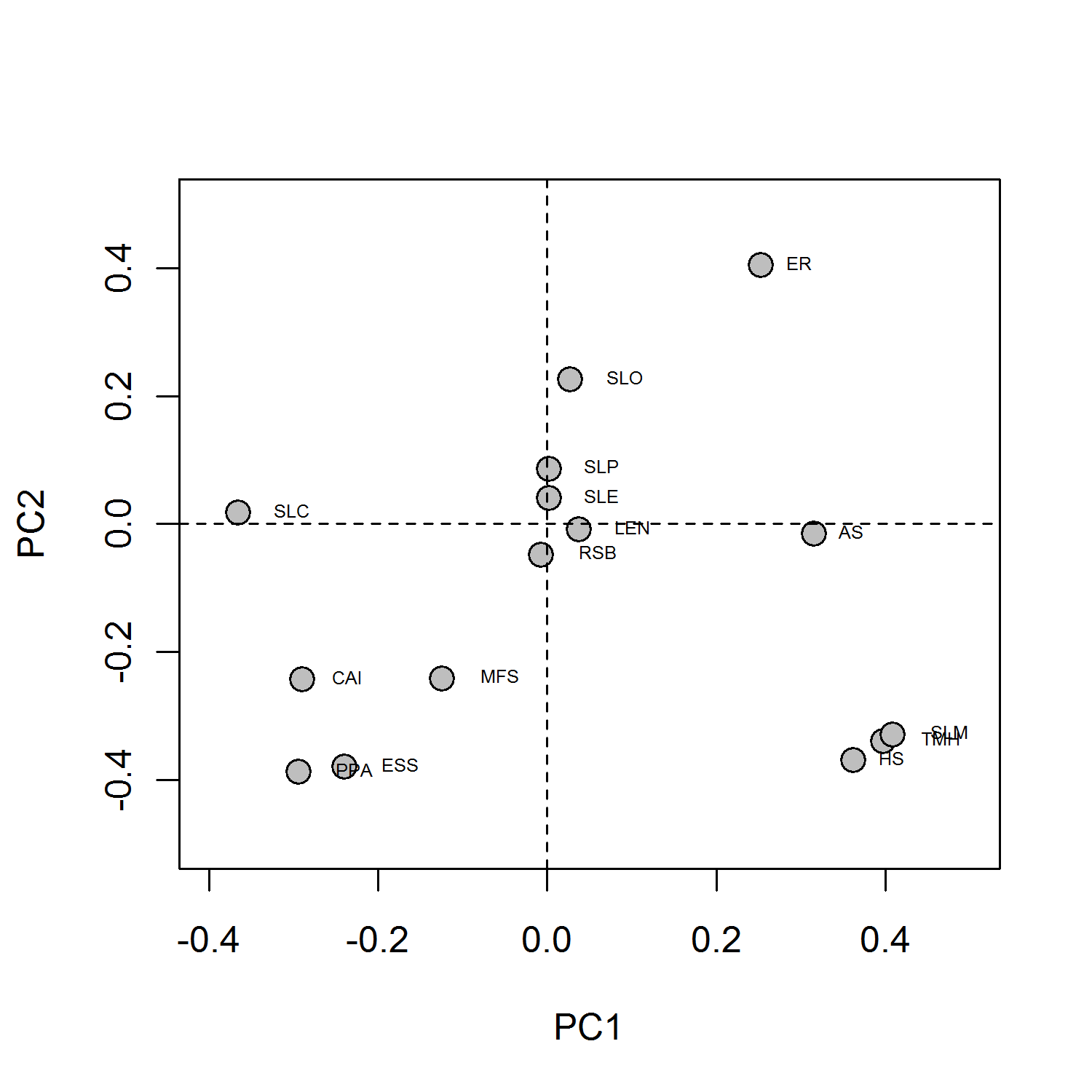 | Bthe  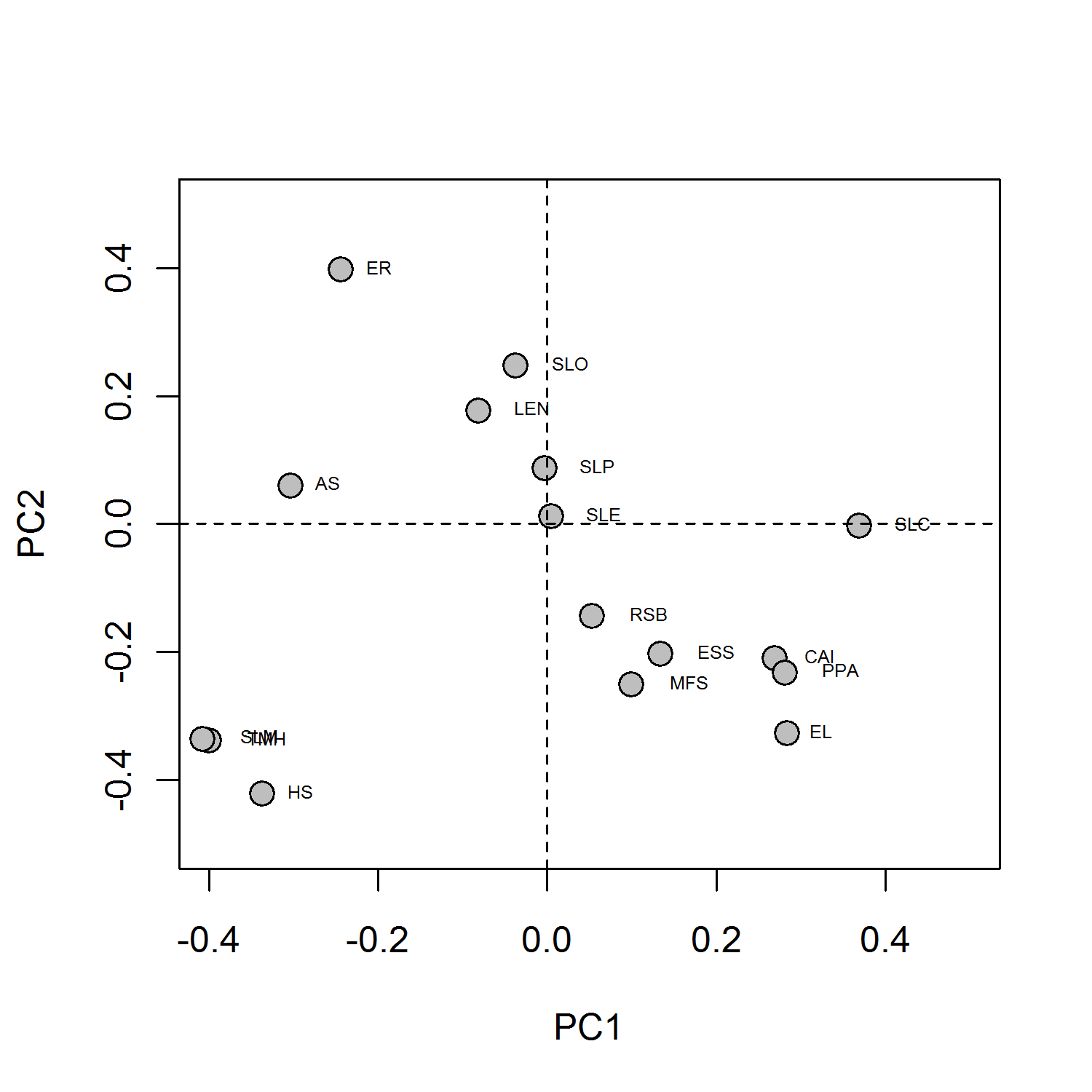 | Ccre  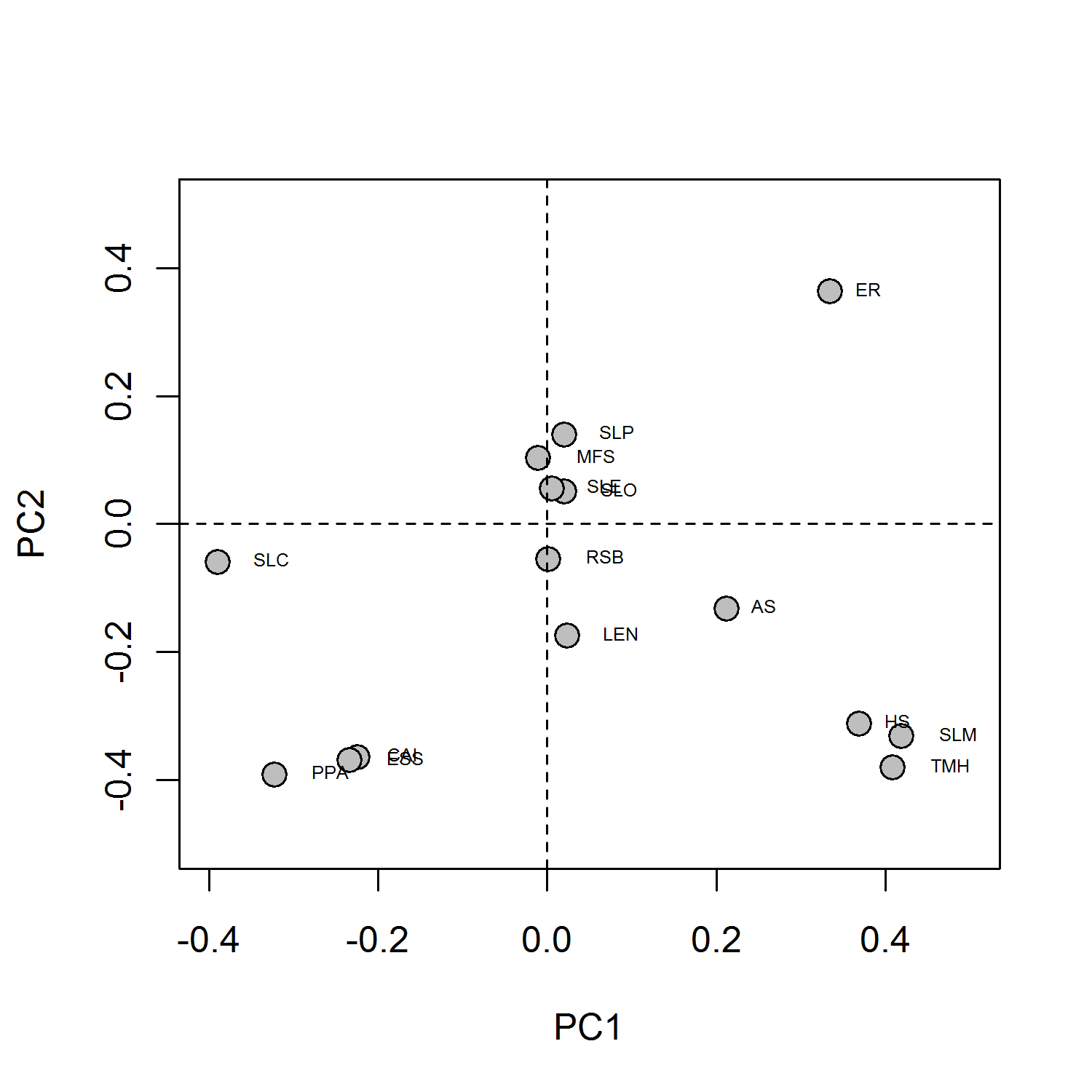 |
| Ecol  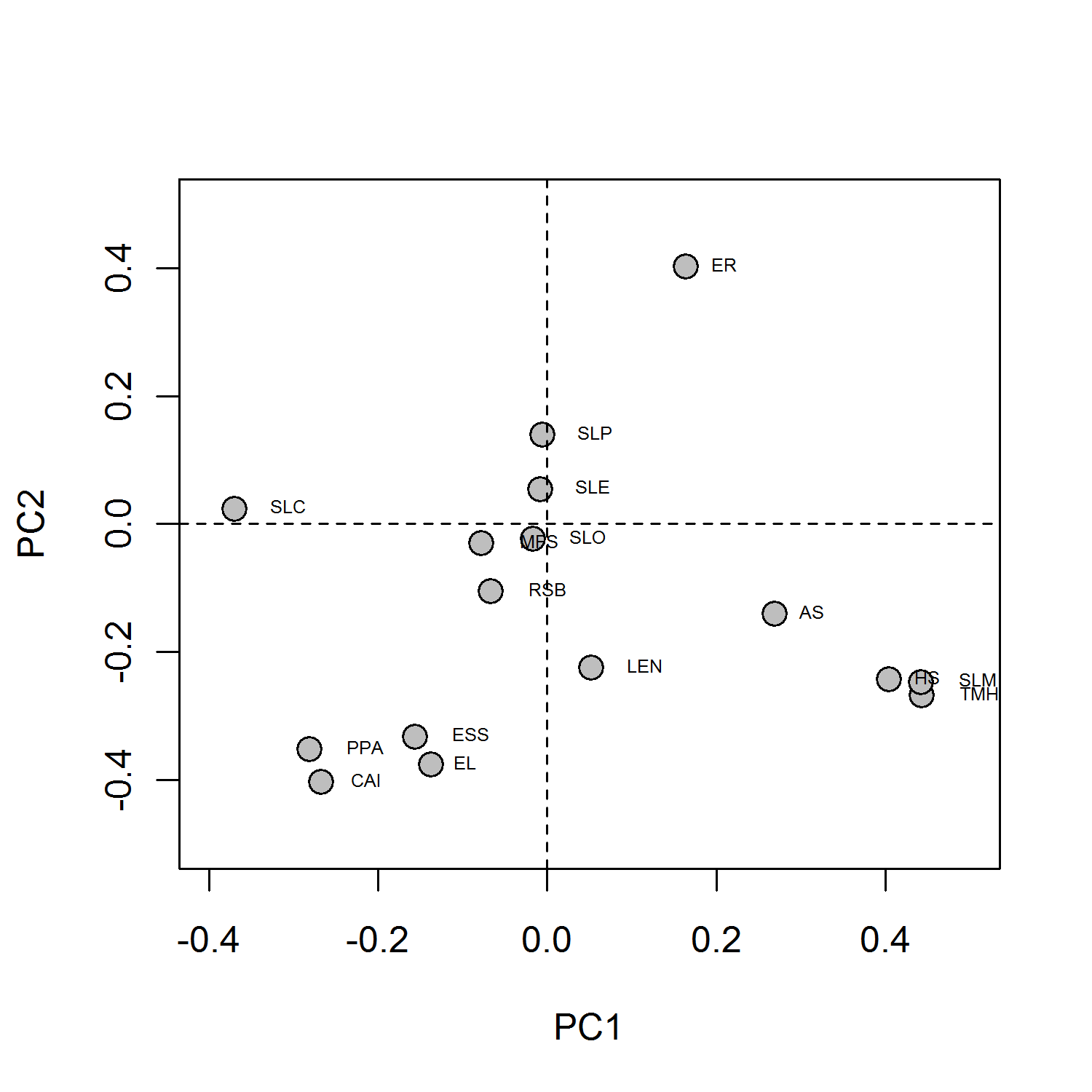 | Fnov  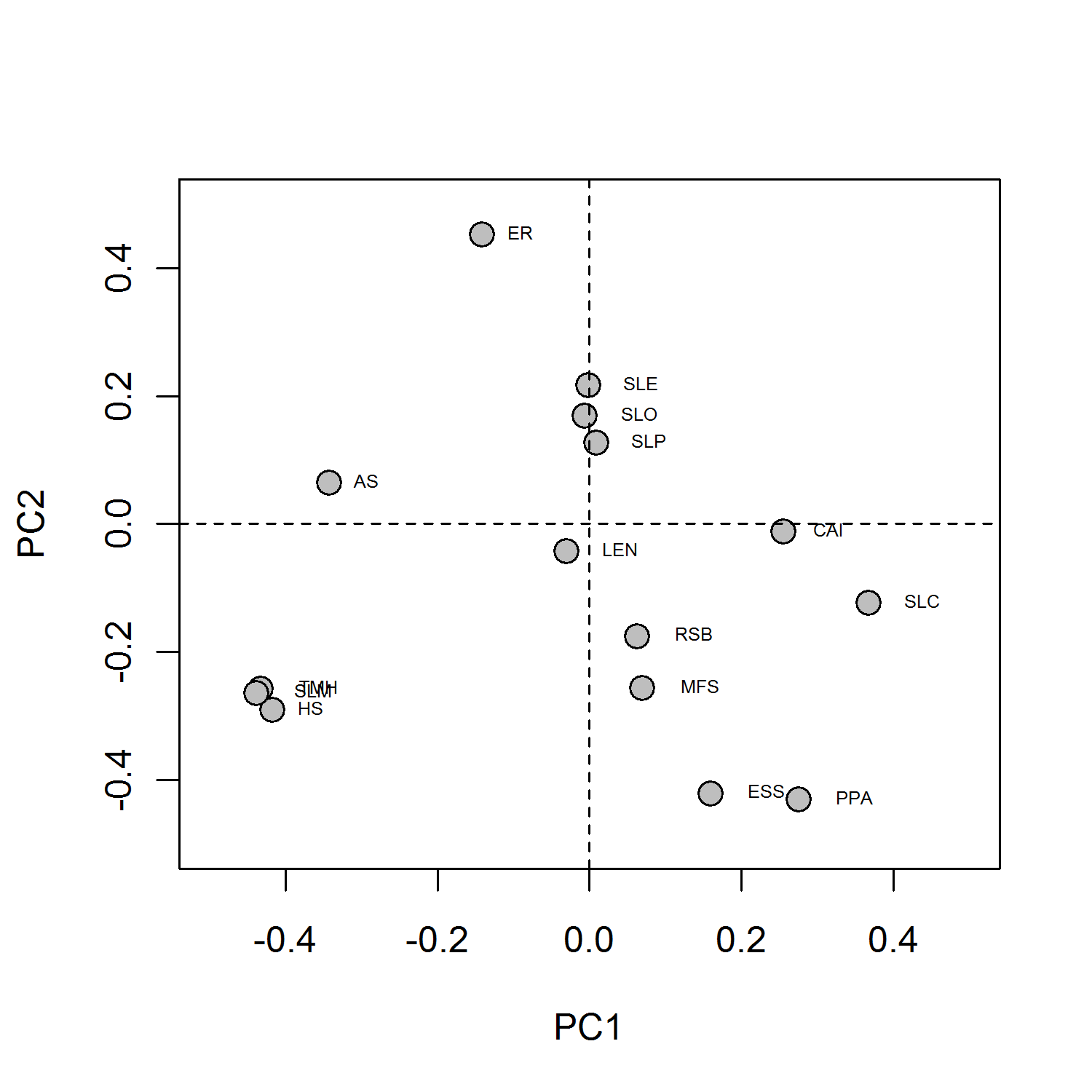 | Hinf  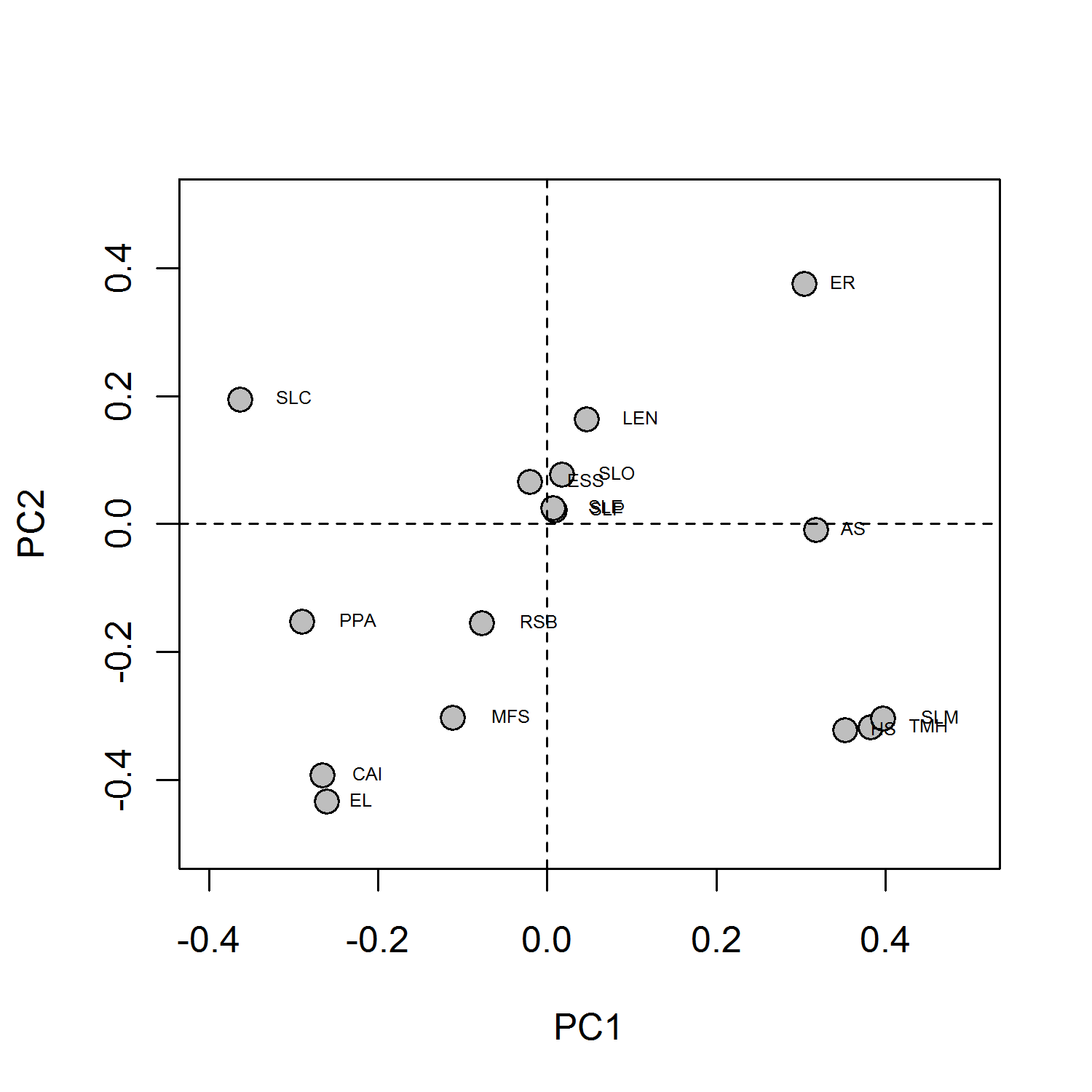 |
| Hpyl  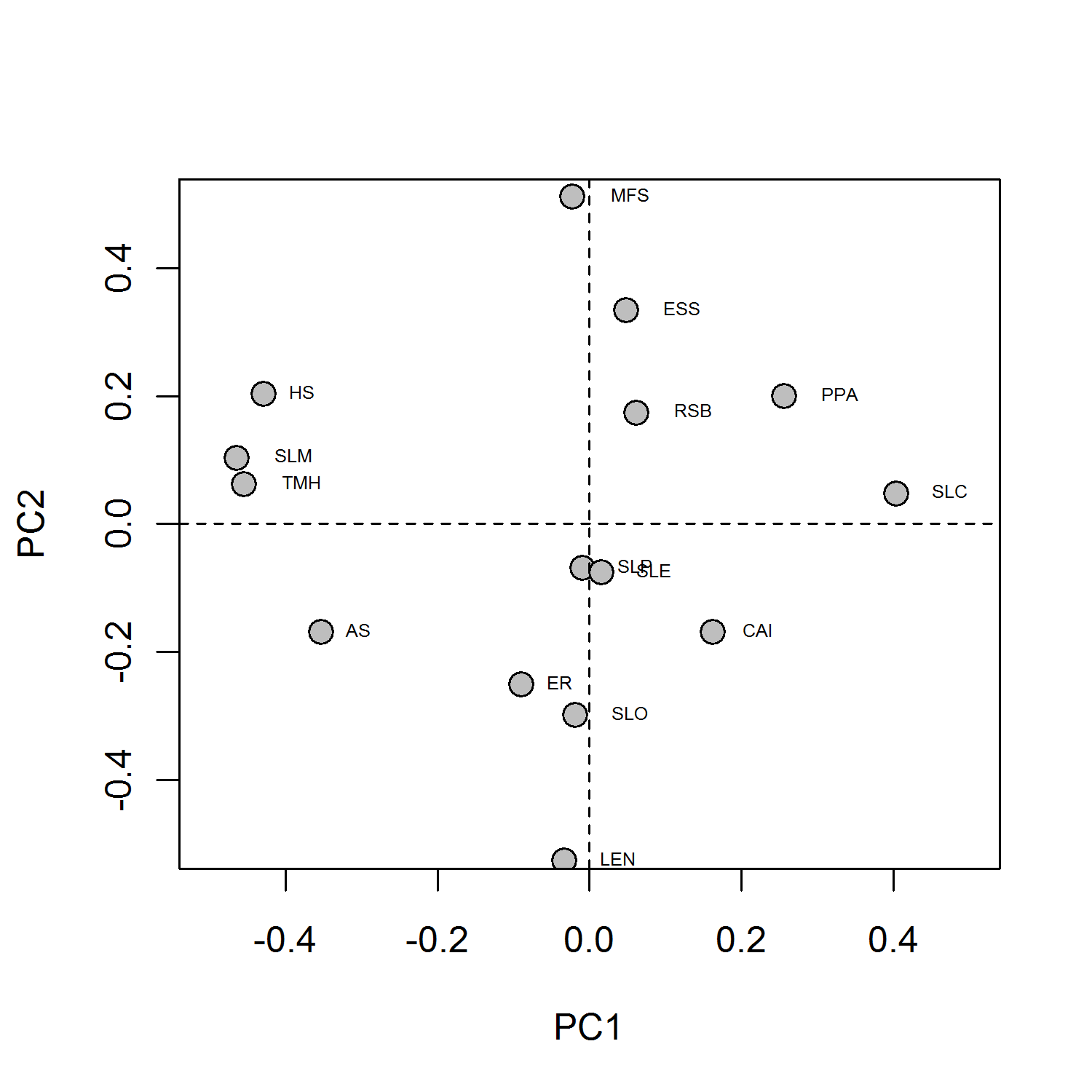 | Paer  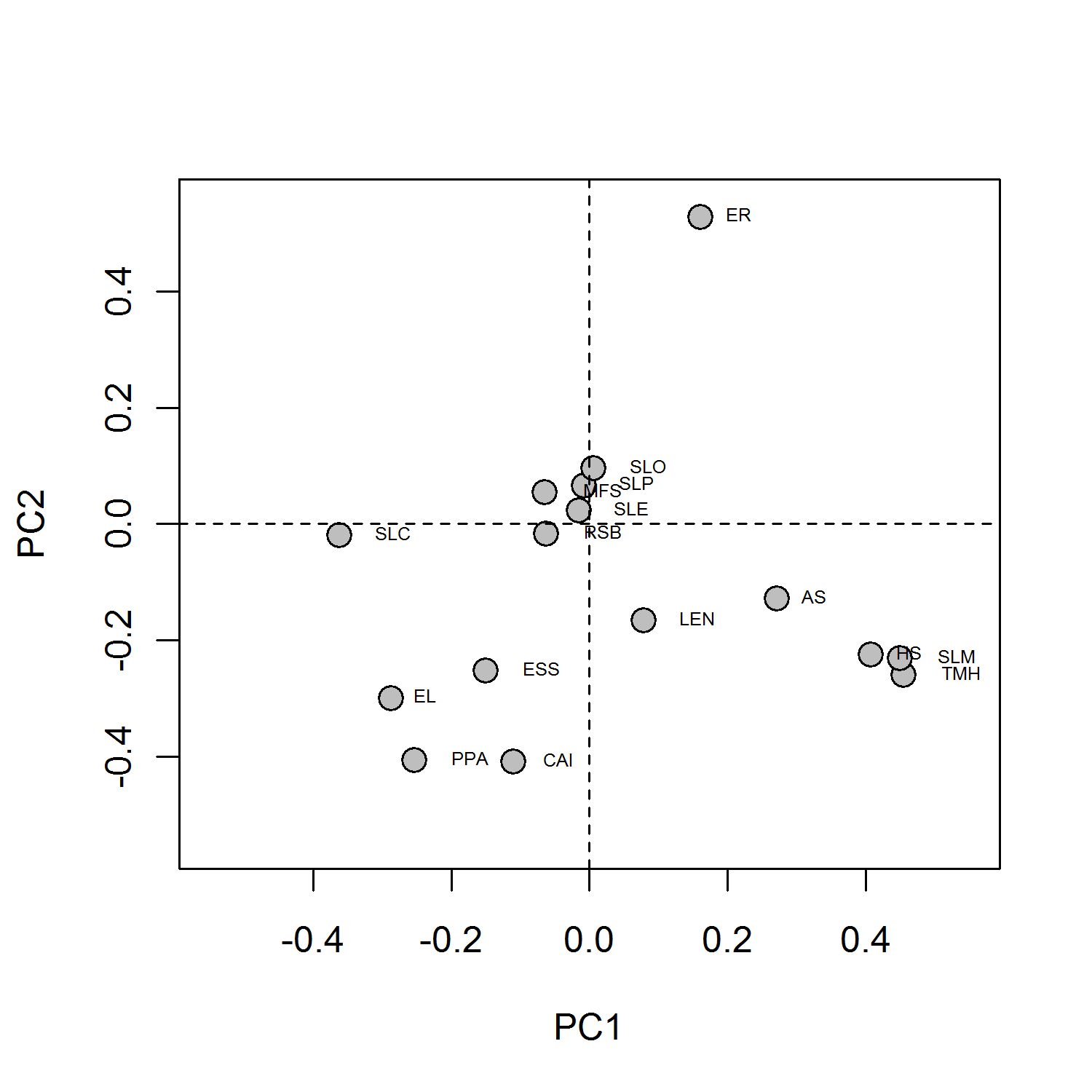 | Pgin  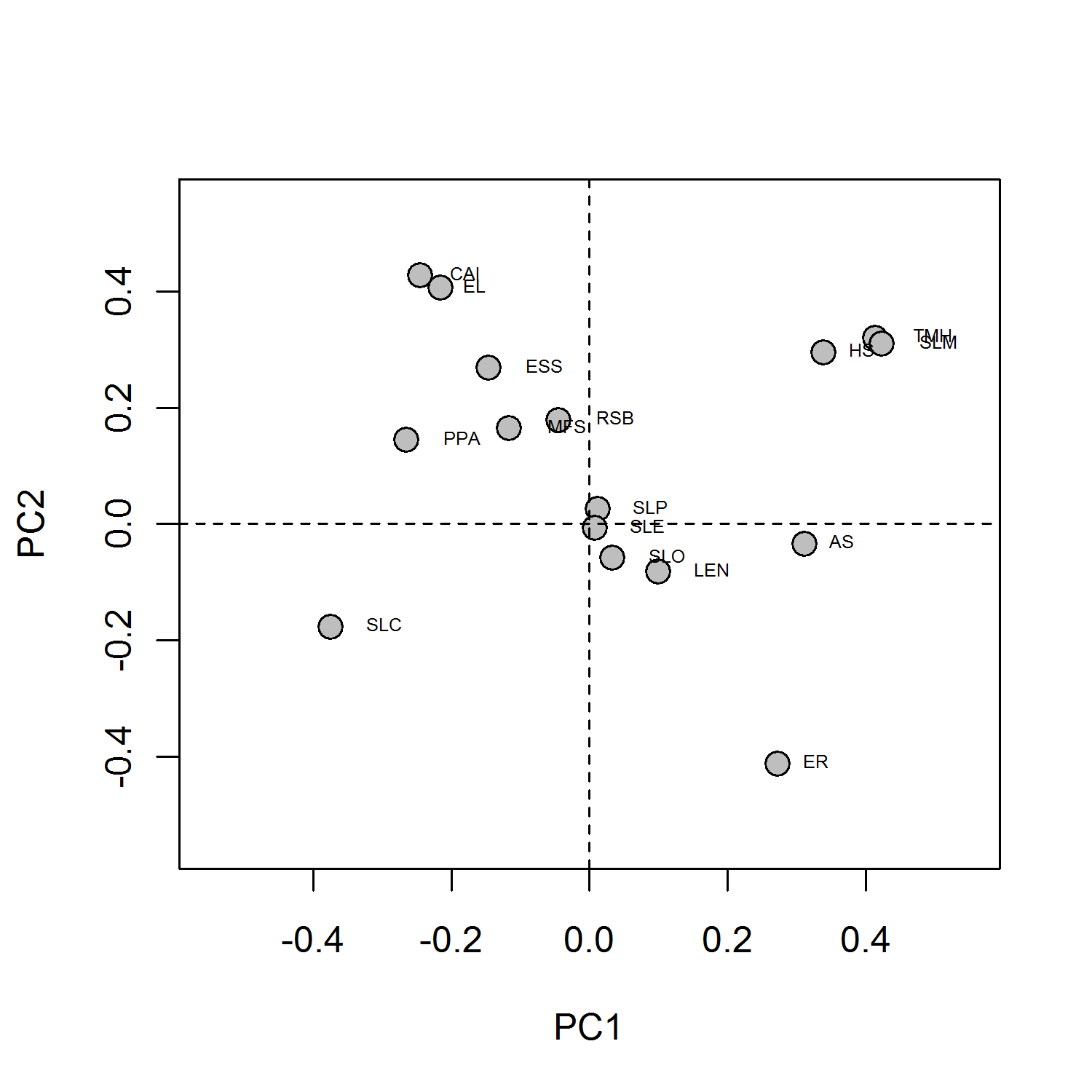 |
| Styp  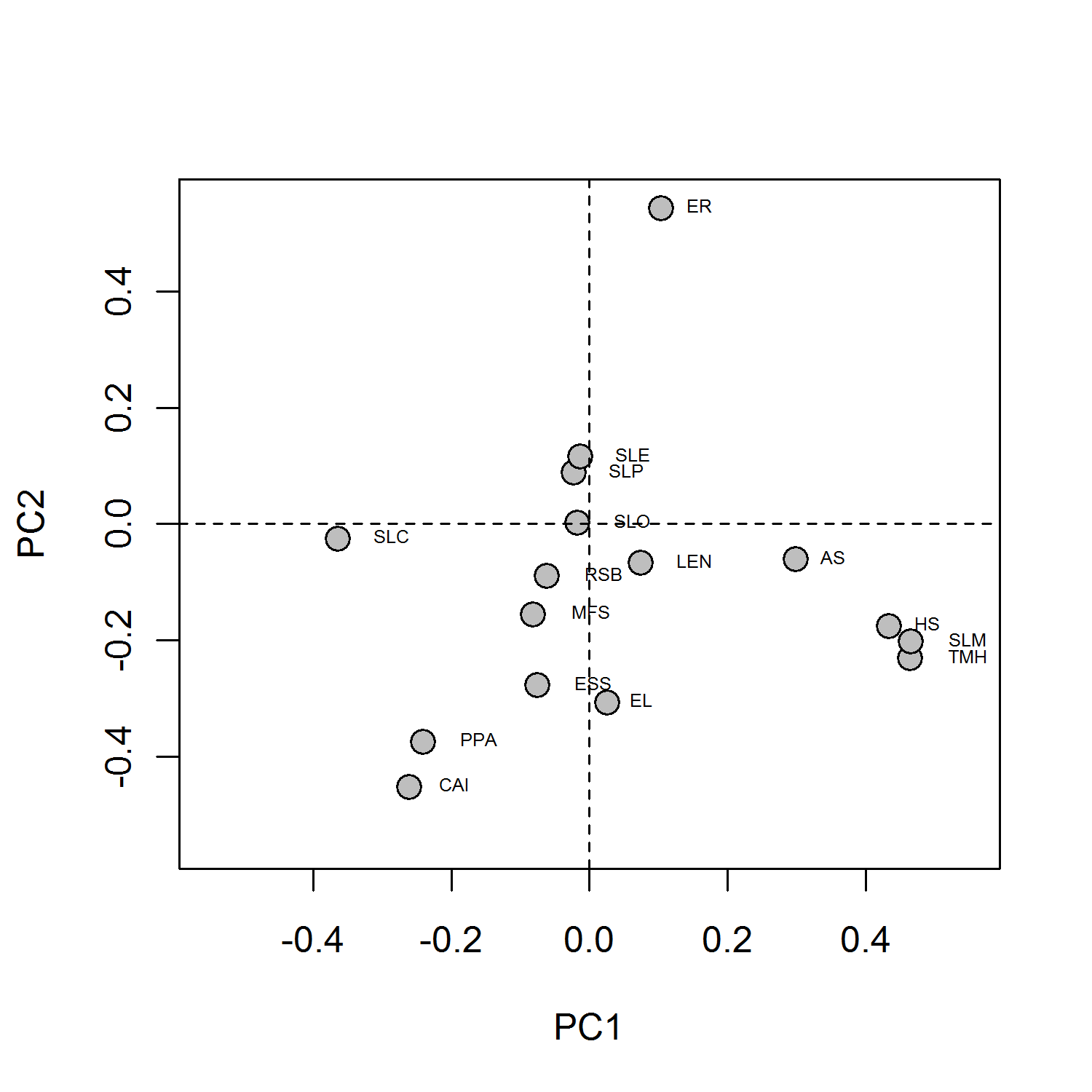 | Vcho  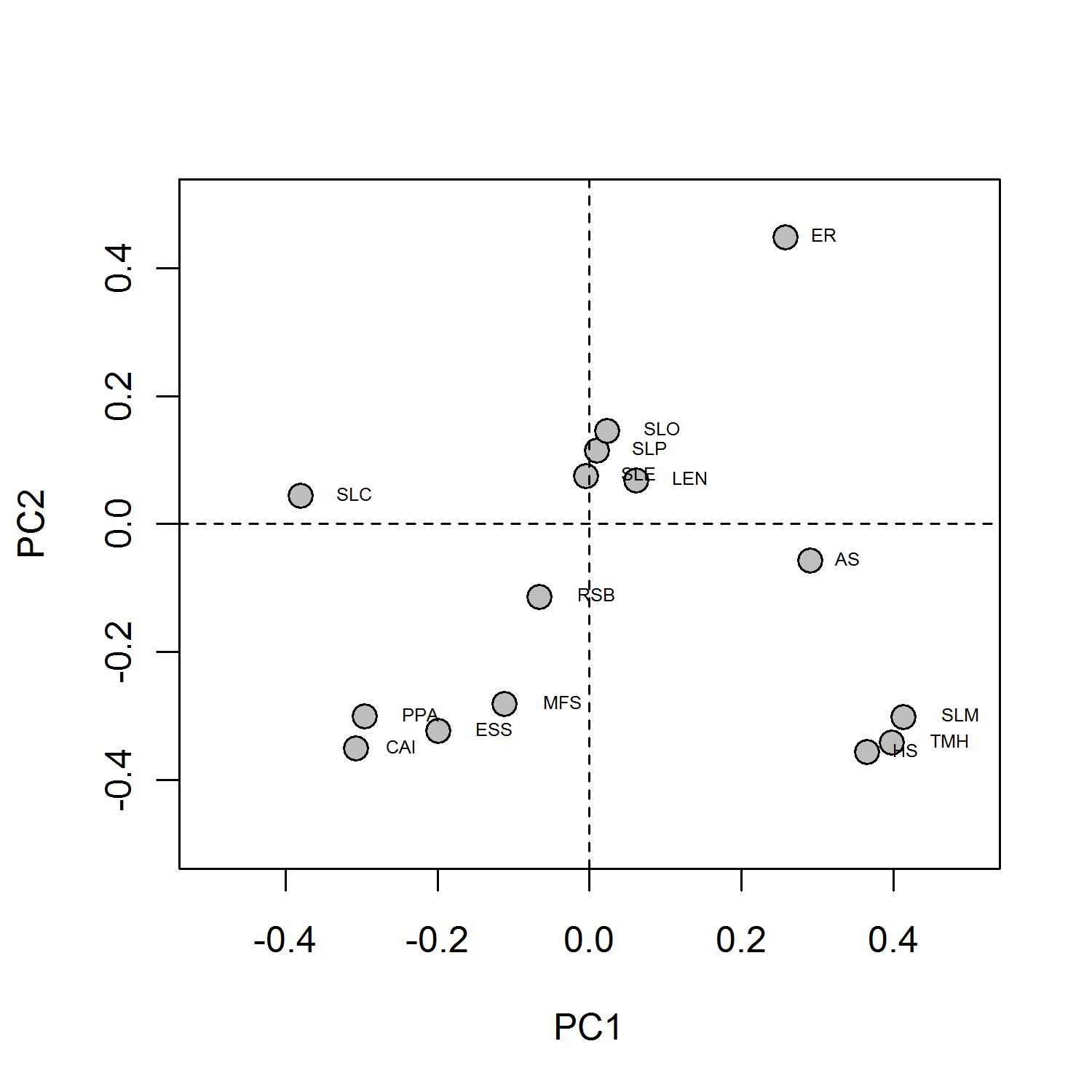 |  |
| Gram-positive organism | | |
| Bsub  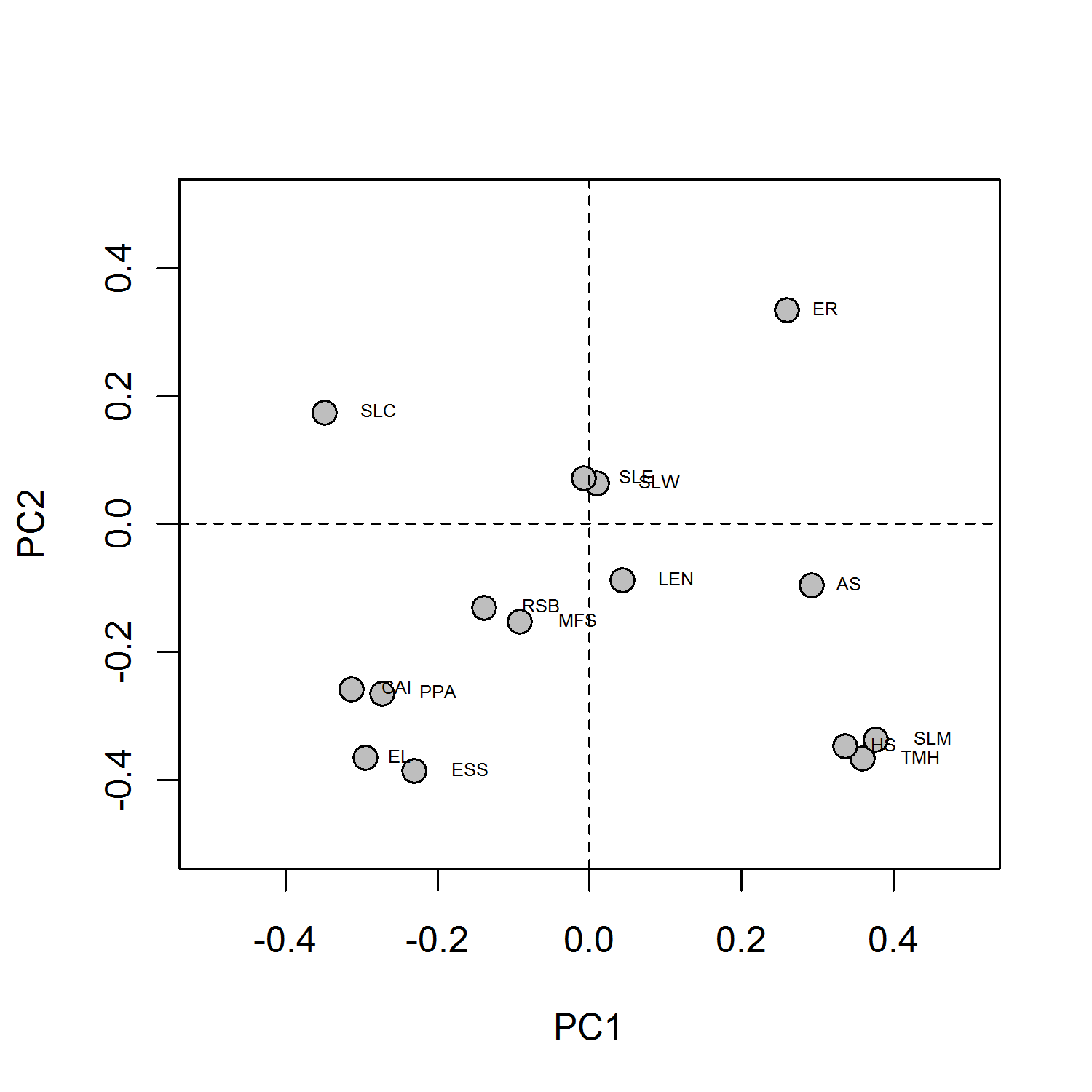 | Mgen  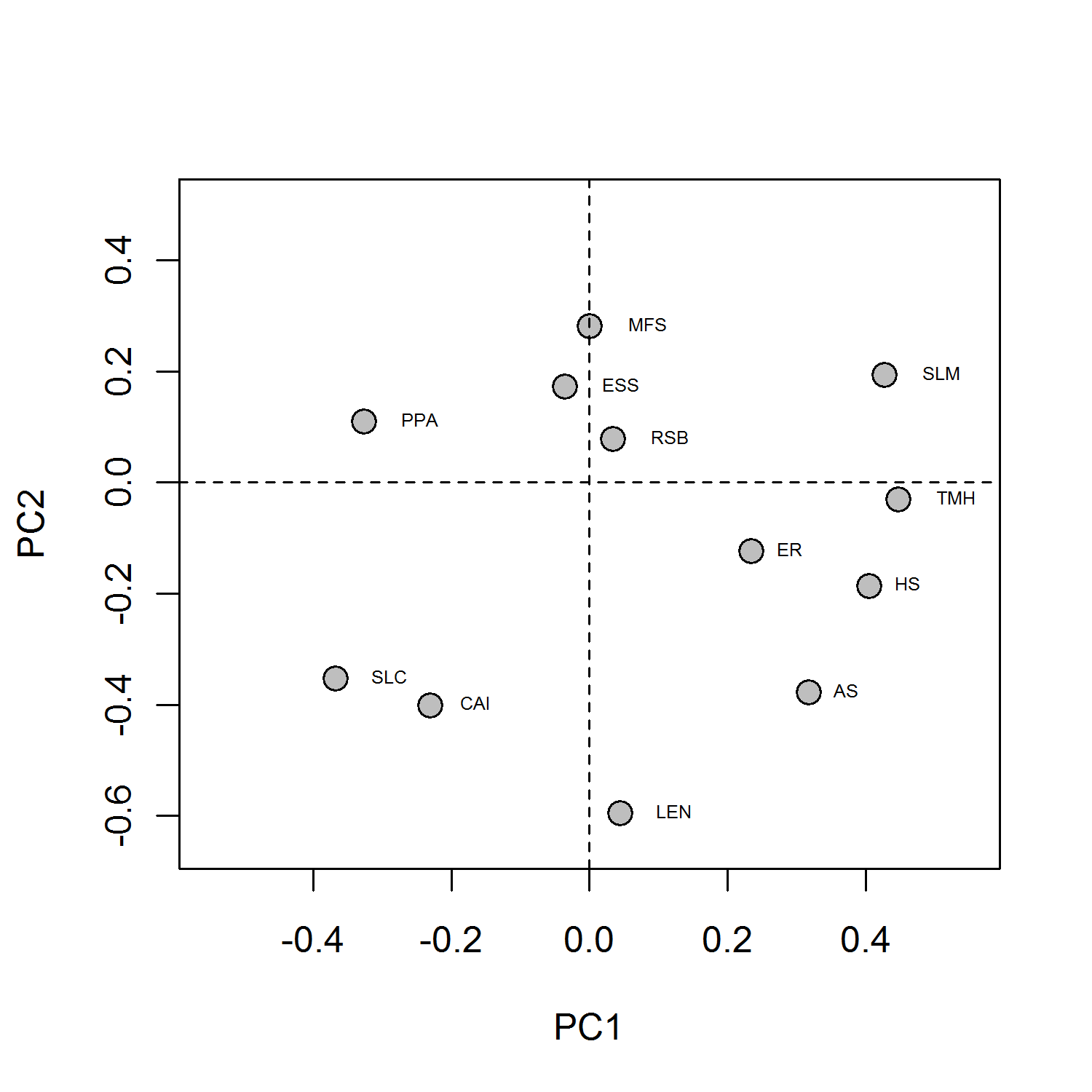 | Mpul  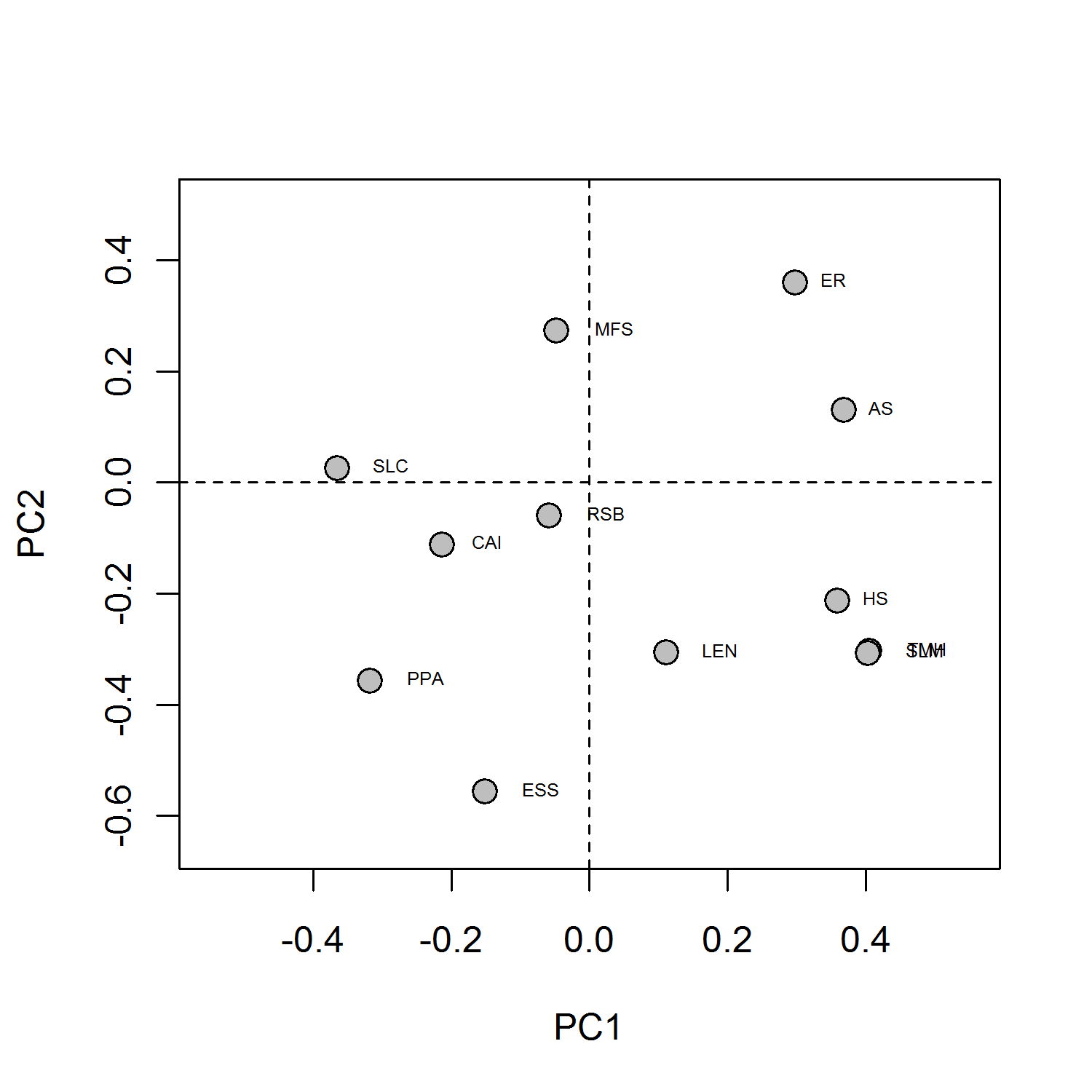 |
| Mtub  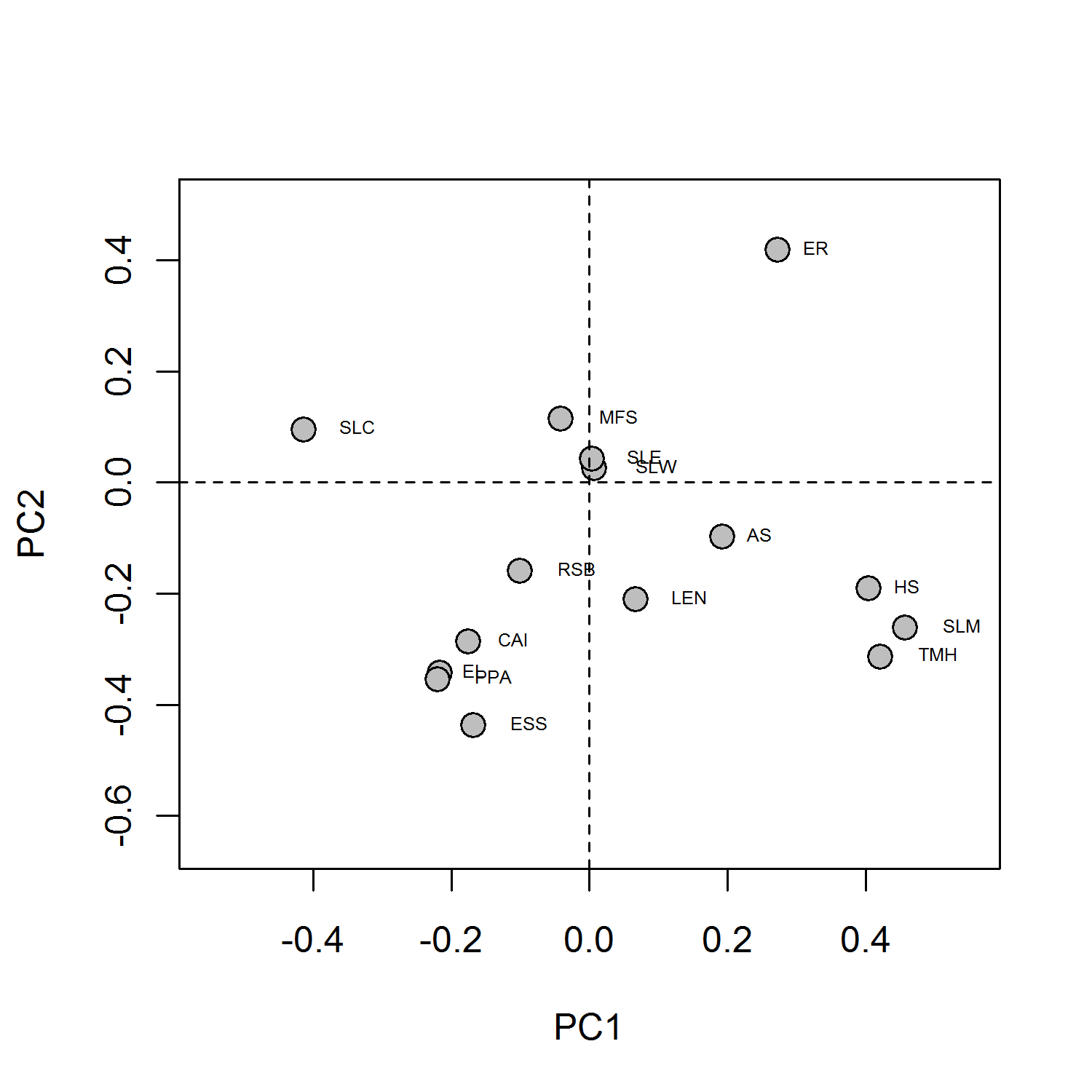 | Spne  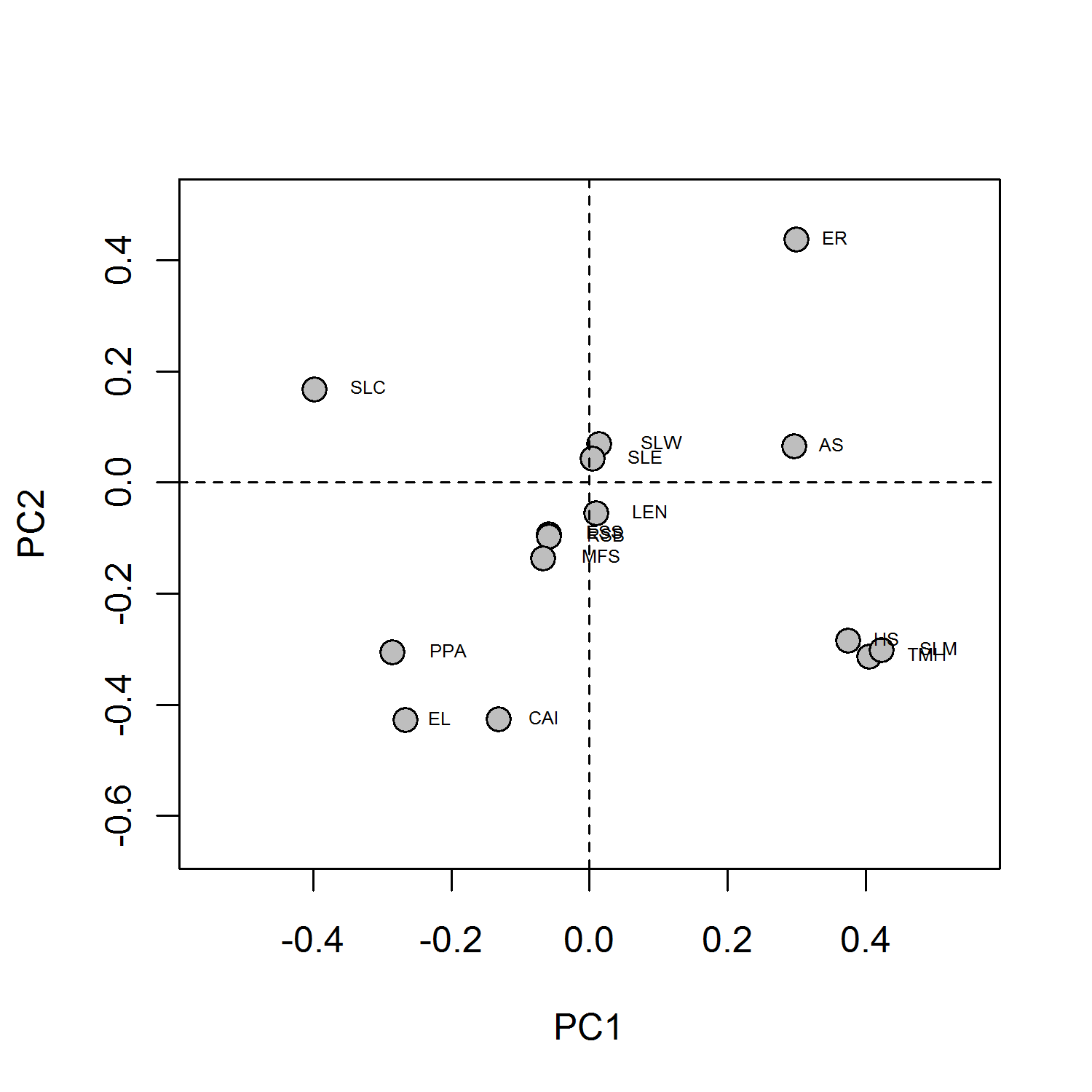 | Ssan  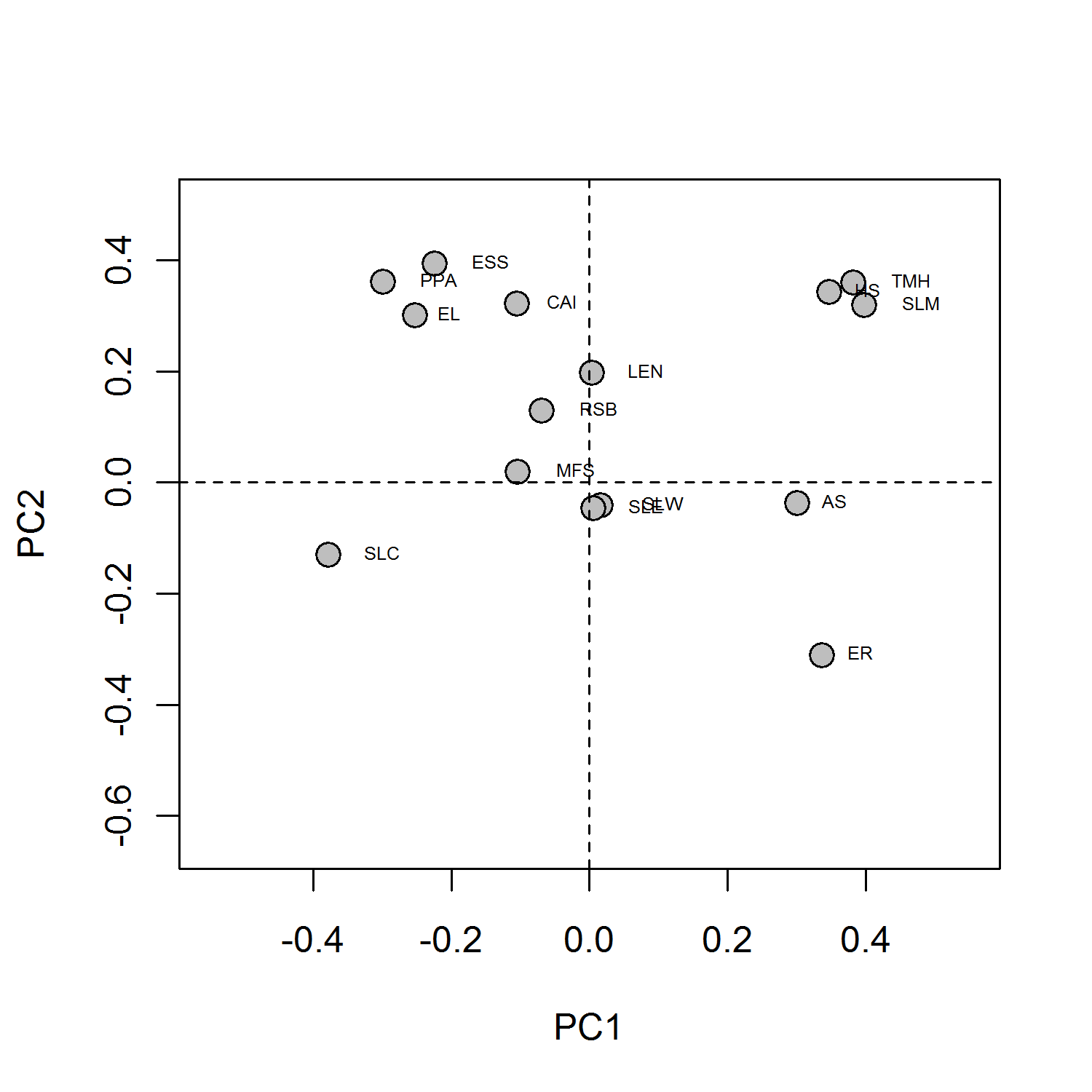 |
